# Systematic Mapping of Worldwide Research on Metabolic Dysfunction-Associated Steatotic Liver Disease (MASLD) and Metabolic Dysfunction-Associated Steatohepatitis (MASH)

**DOI:** 10.1007/s10620-025-09498-7

**Published:** 2025-10-25

**Authors:** Melissa Lou Silva, Renan Prado

**Affiliations:** 1 Department of Radiology, Liver Imaging Group, University of California San Diego, San Diego, USA; 2 Internal Medicine Department, Cleveland Clinic, 9500 Euclid Avenue, Cleveland, OH USA

**Keywords:** MASLD/MASH, Bibliometric analysis, Disparities, Global research trends

## Abstract

**Background and Aims:**

Metabolic dysfunction-associated steatotic liver disease (MASLD) is the most prevalent chronic liver disease worldwide, affecting up to 38% of adults and nearly two-thirds of patients with type 2 diabetes, of whom one-third develop metabolic dysfunction-associated steatohepatitis (MASH). Although only a minority progress to cirrhosis or hepatocellular carcinoma, the absolute burden is substantial and continues to rise, particularly in Asia and Latin America. While population-level disparities are well documented, little is known about inequities within the scientific literature itself. We aimed to systematically map global MASLD/MASH research to identify patterns in productivity, geographic distribution, authorship, and disparities.

**Methods:**

We analyzed publications from 1980 to 2024 in the Web of Science Core Collection (extraction May 15, 2025). After excluding meeting abstracts, 36,268 records were included. Using the *Bibliometrix* R package, we evaluated annual production, country and institutional contributions, authorship patterns, and journal sources. Disparity analyses focused on the 1000 most cited articles, assessing country contributions, first-author sex, and journal representation.

**Results:**

Analysis of the 1000 most cited MASLD/MASH publications revealed rapid growth since the early 2000s, peaking at over 60 papers annually between 2010 and 2020, with an apparent recent decline due to citation lag. The United States dominated both output and citations, far surpassing other countries. Hepatology, Journal of Hepatology, and Gastroenterology accounted for most influential publications. Leading contributors included two authors, with 50 articles each. Striking gender disparities emerged: only 3% of top 100 papers had female first authors. Overall, research remains concentrated geographically, institutionally, and by author, with persistent inequities in authorship representation.

**Conclusions:**

MASLD/MASH research is highly concentrated by geography, journals, and authorship, with pronounced gender disparities. Expanding inclusivity and fostering international collaborations are essential to advance this field.

## Introduction

Metabolic dysfunction-associated steatotic liver disease (MASLD) is now the most common chronic liver disease worldwide, affecting up to 38% of adults and as many as 65% of individuals with type 2 diabetes; 32% of this subgroup also have metabolic dysfunction-associated steatohepatitis (MASH). While only a minority progress to cirrhosis, liver failure, or hepatocellular carcinoma, the vast number of affected patients creates a major healthcare burden. Data from the Global Burden of Disease Study 2021 show rising prevalence, incidence, and disability-adjusted life-years (DALYs) over the past three decades, with the sharpest increases in China and India. The burden peaks at intermediate levels of socioeconomic development, and from 1990 to 2021 MASLD-related DALYs more than doubled, from 1.69 million to 3.67 million [1].

MASLD has emerged as the predominant subtype of steatotic liver disease, representing approximately 75–95% of all cases in both primary care and tertiary settings. Its prevalence in the general adult population ranges from 25 to 39%, depending on the diagnostic modality—underscoring the critical need to consolidate and analyze the expanding body of scientific knowledge on MASH/MASLD [2–4].

Key risk factors for MASLD and MASH include male sex, obesity (especially central adiposity), diabetes, hypertension, dyslipidemia, and sedentary lifestyle, whereas non-Hispanic Black individuals show lower prevalence [2, 4]. Due to it, longitudinal projections suggest that the burden of MASLD and MASH will continue to rise, with up to 101 million MASLD cases and 27 million MASH cases anticipated in the US by 2030 [3, 5].

Disparities in MASLD and MASH are influenced not only by metabolic, genetic, and environmental factors but also by race/ethnicity, socioeconomic status, and access to care. In the U.S., prevalence is highest among Hispanics, intermediate in non-Hispanic Whites, and lowest in non-Hispanic Blacks, differing metabolic risks. Socioeconomic inequities further amplify disease burden, as lower-income populations face higher rates of obesity and diabetes but reduced access to screening and early intervention, leading to delayed diagnosis and worse outcomes [6, 7]. Globally, the burden is rising most rapidly in regions undergoing urbanization and dietary transitions, particularly in Asia and Latin America [1].

While these population-level disparities in MASLD/MASH are well documented [8–10], much less is known about disparities in the scientific literature itself. Gaining a comprehensive understanding of the cumulative body of research on MASLD/MASH—and mapping the distribution of subject matter experts across specialties, institutions, and geographic regions—are essential steps toward building a credible, inclusive, and merit-based multidisciplinary partnership of experts in this field.

## Methods

**Fig. 1:**
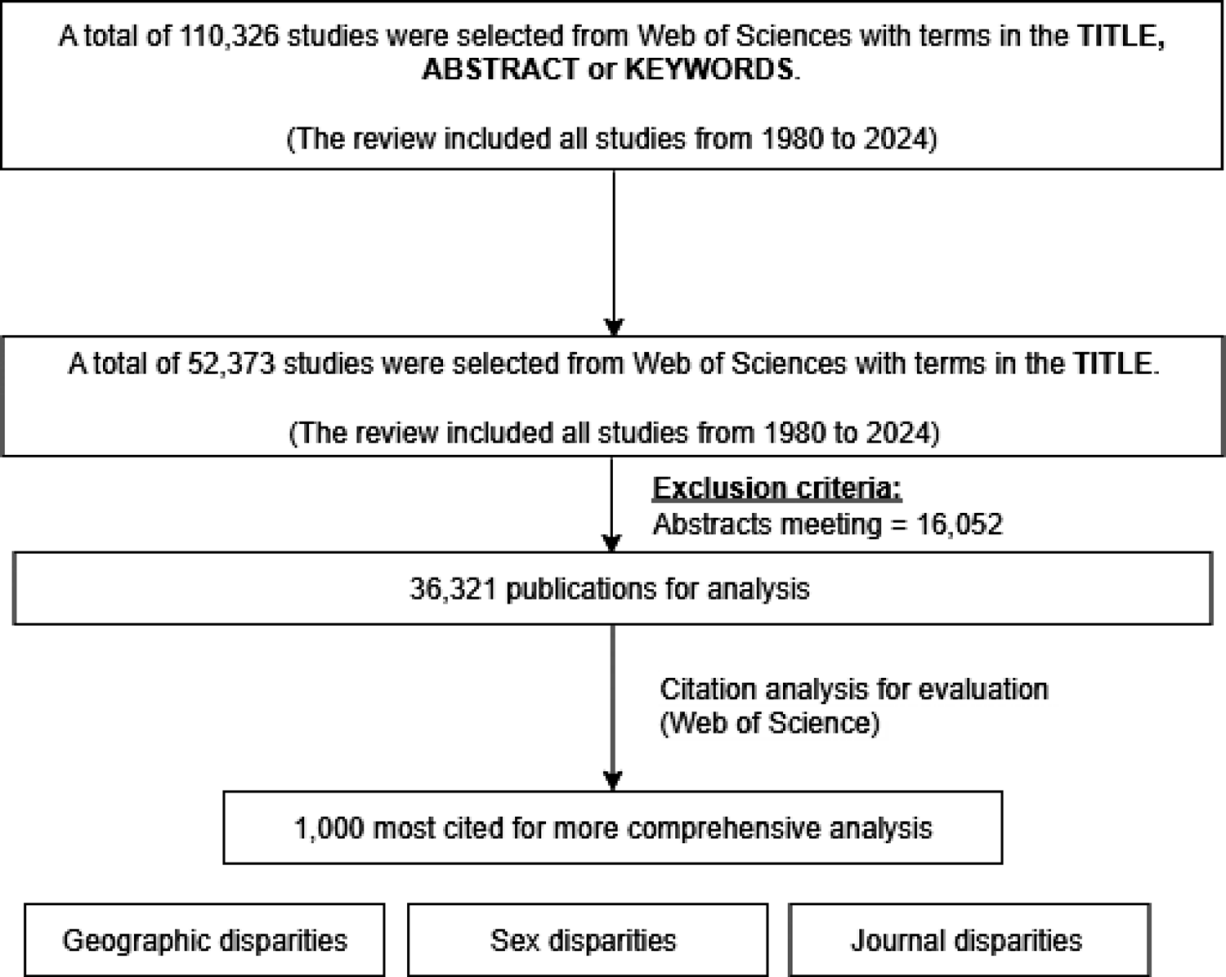
Flow diagram of study methodology

We conducted a bibliometric analysis using the Web of Science Core Collection (data extracted on May 15, 2025). All publications from 1980 to 2024 containing the terms *NASH*, *MASH*, *NAFLD*, *MASLD*, *MAFLD*, or related variants in the title were included in this review. This represents a subset of a broader search strategy that also encompassed the title, abstract, and keywords. (Fig. 1).

After excluding meeting abstracts (*n* = 16,052), a total of 36,321 publications were retained for analysis. Using the Bibliometrix R package, we evaluated annual scientific production, geographic and authorship distribution, and journal characteristics. For disparity analyses, we focused on the 1000 most cited studies, examining geographic representation, first-author sex distribution, and journal-related disparities.

## Results

### Annual Volume of Publications

**Fig. 2:**
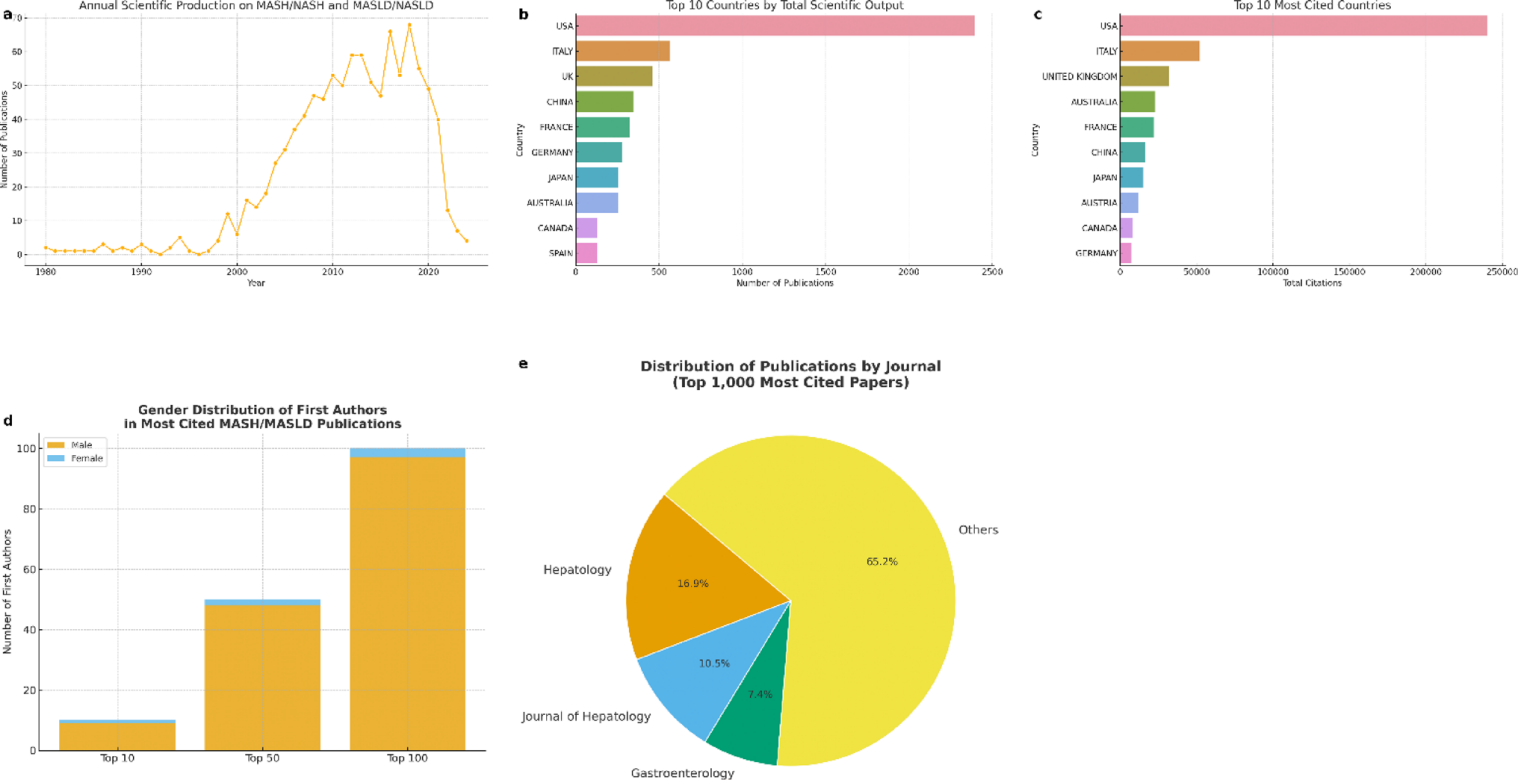
Key features of contemporary MASLD/MASH research: **a** annual scientific production; **b** top 10 countries by total publications; **c** top 10 countries by total citations; **d** gender distribution of first authors; **e** most relevant journals

Between 1980 and the early 2000s, annual publications on MASH/NASH and MASLD/NASLD remained very limited, rarely exceeding 10 articles per year. A rapid growth phase began in the early 2000s, with the number of studies increasing steadily and peaking between 2010 and 2020. This surge reflects the growing recognition of fatty liver disease as a major public health concern and the corresponding expansion of research activity. After 2020, a sharp decline is observed in the curve. However, this apparent reduction must be interpreted with caution: our analysis focused on the 1000 most cited publications, which naturally favors older, well-established studies. As a result, the most recent articles may be underrepresented due to insufficient time to accumulate citations, and the drop in production does not necessarily reflect a true decrease in research activity. (Fig. 2a).

### Types of Study

Among the publications analyzed, original research articles represented the majority, accounting for 65.6% of all records. Review articles followed with 12.7%, while editorial materials and letters contributed 5.1% and 4.9%, respectively. The remaining 11.8% of publications were grouped as other, encompassing conference proceedings, book reviews, corrections, and miscellaneous items. Overall, this distribution highlights a strong predominance of original research, with supplementary contributions from reviews and editorials shaping the broader scientific discourse.

Also, evaluating all publications, the largest proportion belonged to the field of Gastroenterology and Hepatology, comprising 27.6% of all records. This was followed by Endocrinology and Metabolism (8.0%), Pharmacology and Pharmacy (7.8%), and Biochemistry and Molecular Biology (7.3%), reflecting the strong intersection between metabolic and molecular research domains. Additional major categories included General Internal Medicine (6.7%), Nutrition and Dietetics (6.6%), and Experimental Medicine (5.1%), highlighting the multidisciplinary nature of literature.

### Geographical Location

Figure 1b shows the countries with the highest total scientific output in MASH/NASH and MASLD/NAFLD research. The United States is by far the most prolific, followed by Italy and the United Kingdom. Other leading contributors include China, France, Germany, Japan, Australia, Canada, and Spain, highlighting the global interest in fatty liver disease research across North America, Europe, and Asia–Pacific regions.

Figure 1c presents the distribution of total citations, reflecting research impact. Again, the United States dominates, with nearly 240,000 citations—an order of magnitude higher than other countries. Italy and the United Kingdom follow, with approximately 52,000 and 32,000 citations, respectively. Other countries such as Australia, France, China, Japan, Austria, Canada, and Germany also appear among the top 10, but with considerably fewer citations compared to the United States.

Together, these findings reveal that while multiple countries actively contribute to the MASH/MASLD field, the United States not only produces the largest volume of studies but also garners the greatest global impact, underscoring its leadership role in shaping the literature. (Fig. 2b, c).

In our analysis, each publication was assigned to the country of the corresponding author’s affiliation, a standard bibliometric practice that prevents double-counting in multinational studies. Nevertheless, we acknowledge that many highly cited MASLD publications arise from international collaborations involving multiple institutions and countries, which can limit the interpretability of strictly country-level analyses.

**Fig. 3:**
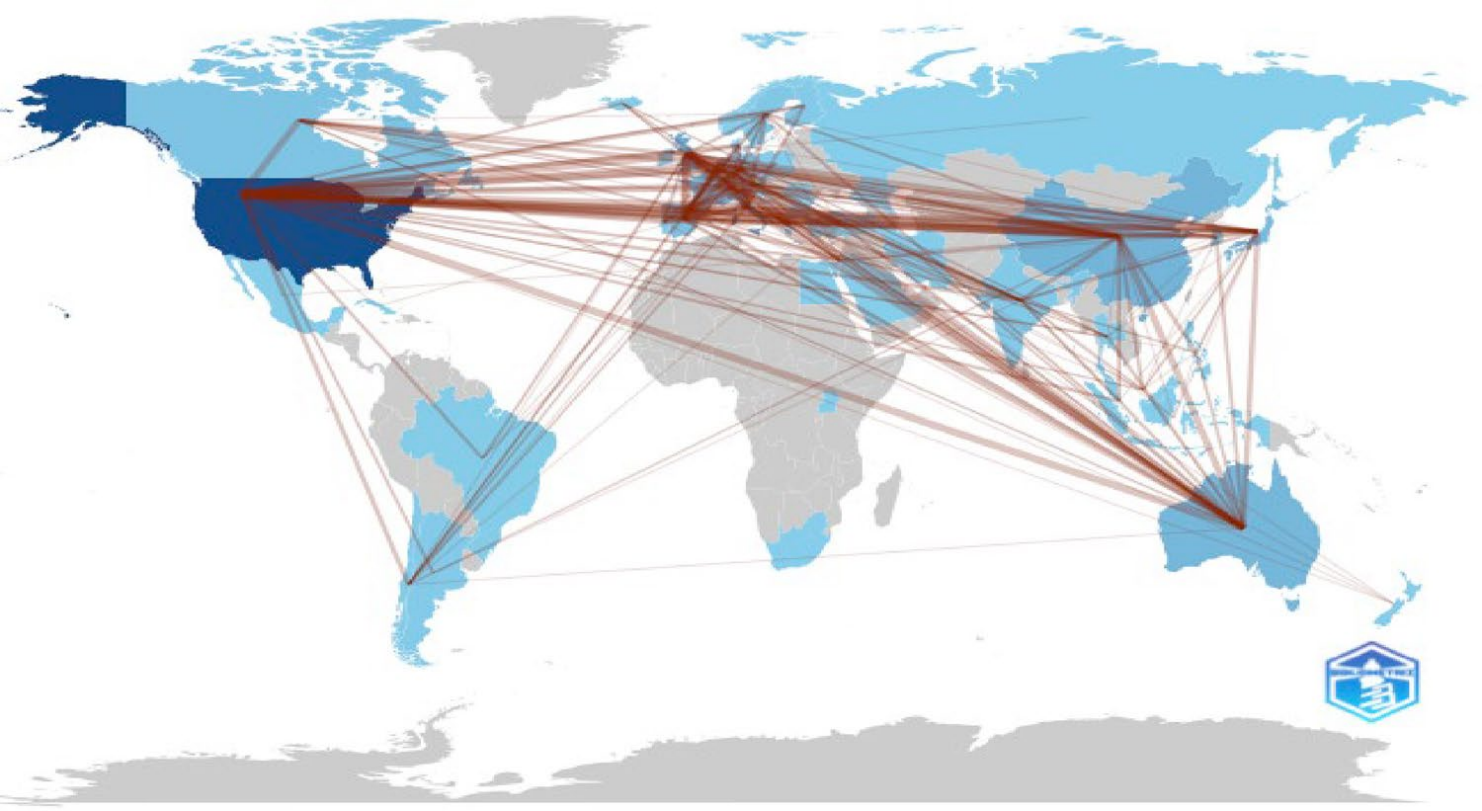
Figure showing collaboration between authors/countries, highlighting disparities

To provide a more balanced global perspective, we conducted an additional region-based aggregation, grouping countries into six major regions—North America, Central/South America, Europe, Asia, Oceania, and Africa—and accounting for all collaborative authorships. Among the 6,087 authors analyzed, North America contributed the largest share (2,531; 41.6%), followed by Europe (2,261; 37.1%) and Asia (1,020; 16.8%). Contributions from Oceania represented 4.2%, while Central/South America and Africa remained markedly underrepresented, accounting for only 1.5% and 0.3% (Fig. 3).

### Journals/Authors Contribution

For journals, *Hepatology* is the clear leader, with 169 publications, followed by the *Journal of Hepatology* (105) and *Gastroenterology* (74). Other high-impact outlets such as *Nature Reviews Gastroenterology & Hepatology*, *Gut*, and the *American Journal of Gastroenterology* also feature prominently. This distribution underscores that the bulk of influential work in the field has been concentrated within premier hepatology and gastroenterology journals, ensuring high visibility among liver and digestive disease specialists. (Table 1, Fig. 2e).

**Table 1:** Leading journals and authors among the top 1000 most cited MASH/MASLD publications

Journal	Articles, *N* (%)
Hepatology	169 (16.9%)
Journal of Hepatology	105 (10.5%)
Gastroenterology	74 (7.4%)

Authors	Articles, *N* (%)
Loomba R	50 (5.0%)
Sanyal AJ	50 (5.0%)
Harrison SA	37 (3.7%)

The most prolific contributing authors. Rohit Loomba and Arun J. Sanyal each lead with 50 publications, reflecting their pivotal roles in advancing the field of fatty liver disease. They are closely followed by Stephen A. Harrison (37 articles), Anna Mae Diehl (34), and Elizabeth M. Brunt (33), all internationally recognized leaders in hepatology. The presence of these highly influential investigators reflects both the depth of expertise and the collaborative nature of research in this domain. (Table 1).

Together, these findings demonstrate that a relatively small group of high-impact journals and leading investigators drive most of the highly cited work in MASH/MASLD, consolidating both academic influence and research visibility in this area.

### Sex Disparities

Among the most influential publications, male authors were overwhelmingly predominant. In the 10 most cited articles, only one was led by a female first author. This pattern persisted in the top 50, where just 2 female first authors were identified, representing only 4%. In the top 100 most cited publications, male authors accounted for 97% of first authorships, with females representing only 3%. These findings underscore a significant gender imbalance, with landmark works in the MASH/MASLD literature largely driven by male investigators. (Fig. 1d, Table 2).

**Table 2:** Author gender distribution in most cited papers

Range	Male	Female	Total
Top 10	9	1	10
Top 50	48	2	50
Top 100	97	3	100

## Discussion

This bibliometric analysis of MASLD/MASH-related literature reveals marked disparities in global research output, authorship representation, and citation impact. The United States overwhelmingly dominates the field, accounting for most highly cited publications (42.5%)—more than double the percentage observed in broader gastroenterology and hepatology research, where the U.S. contributed 17.4% of total output between 2009 and 2018 [11]. This disproportionate concentration of research leadership mirrors patterns observed across gastroenterology and hepatology bibliometrics, but the gap in MASLD/MASH research appears even more pronounced.

Authorship trends further underscore existing inequities. Among the most cited publications, male investigators accounted for nearly all landmark contributions, with female first authors representing only 1 of the top 10, 2 of the top 50, and 3 of the top 100 most cited papers. This severe underrepresentation contrasts with prior analyses of gastroenterology and hepatology literature. For example, previous studies in major GI journals reported that only 24% of first authors were female [12]. Our findings reveal an even more pronounced gender imbalance, with only 3% of first authors being female among the top 100 most cited MASLD/MASH papers. Comparable disparities have been reported in prior bibliometric analyses of gastroenterology and hepatology, where women represented just 15.1% of first authors among the top 50 most cited articles [8]. However, the absence of global data on the gender distribution of gastroenterologists limits broader comparisons.

Such disparities are not unique to hepatology. Gender imbalances in authorship and citation influence have been well documented across cardiology, surgery, ophthalmology, radiology, and other specialties. Commonly cited contributors include limited access to mentorship, unequal allocation of research funding, and structural barriers to promotion and leadership. In gastroenterology, disparities are often even more pronounced for senior and last authorship positions, where women remain disproportionately absent from higher academic ranks. This imbalance risks perpetuating inequities in visibility, recognition, and influence within the field.

At the journal and author level, our analysis demonstrates that a small cluster of high-impact outlets (*Hepatology, Journal of Hepatology, Gastroenterology*) and prolific are disproportionately responsible for shaping the MASLD/MASH literature. While this concentration reflects expertise and consistent scholarly productivity, it also underscores the need for broader diversification of voices and perspectives to avoid research silos and ensure inclusive progress.

Also, it is important to note that, even with increasing migration and globalization, it remains unrealistic to assume that data derived predominantly from populations in North America and Europe—which together account for nearly 80% of global research output—can fully capture the sociocultural, dietary, and lifestyle determinants influencing MASLD in other regions. In contrast, Central/South America and Africa, which together represent approximately one-third of the world’s population but contribute only 1.8% of the literature, remain markedly underrepresented.

Consequently, the generalizability of current evidence must be interpreted with caution, particularly for populations that are systematically underrepresented both in research and in clinical cohorts. We have expanded the discussion to highlight that this study does not aim to change clinical practice directly but rather to reshape how the existing MASLD literature is interpreted, acknowledging its geographic, gender and socioeconomic bias.

## Conclusion

This bibliometric analysis of the 1000 most cited publications on MASH/NASH and MASLD/NAFLD highlights the key structural features of the field. The literature is heavily concentrated within a small number of high-impact journals and shaped by a relatively limited group of prolific investigators. Notably, female first authors remain markedly underrepresented, even among the most influential studies. These findings emphasize the urgent need for more inclusive authorship, broader global participation, and strengthened international collaborations to advance research and effectively address the growing global burden of fatty liver disease.

## Data Availability

No datasets were generated or analysed during the current study.
